# MAMVCL: Multi-Atlas Guided Multi-View Contrast Learning for Autism Spectrum Disorder Classification

**DOI:** 10.3390/brainsci15101086

**Published:** 2025-10-08

**Authors:** Zuohao Yin, Feng Xu, Yue Ma, Shuo Huang, Kai Ren, Li Zhang

**Affiliations:** 1 College of Information Science and Technology & Artificial Intelligence, Nanjing Forestry University, Nanjing 210037, China; yinzuohao@njfu.edu.cn (Z.Y.); huangshuo@njfu.edu.cn (S.H.); lizhang@njfu.edu.cn (L.Z.); 2 School of Clinical Medicine, Jiangsu Health Vocational College, Nanjing 210000, China; mayue@jshvc.edu.cn; 3 School of Mechanical and Electronic Engineering, College of Nanjing Forestry University, Nanjing 210037, China; kairen@njfu.edu.cn

**Keywords:** autism spectrum disorder (ASD), population graph, graph contrastive learning, classification

## Abstract

Background: Autism spectrum disorder (ASD) is a neurodevelopmental condition characterized by significant neurological plasticity in early childhood, where timely interventions like behavioral therapy, language training, and social skills development can mitigate symptoms. Contributions: We introduce a novel Multi-Atlas Guided Multi-View Contrast Learning (MAMVCL) framework for ASD classification, leveraging functional connectivity (FC) matrices from multiple brain atlases to enhance diagnostic accuracy. Methodology: The MAMVCL framework integrates imaging and phenotypic data through a population graph, where node features derive from imaging data, edge indices are based on similarity scoring matrices, and edge weights reflect phenotypic similarities. Graph convolution extracts global field-of-view features. Concurrently, a Target-aware attention aggregator processes FC matrices to capture high-order brain region dependencies, yielding local field-of-view features. To ensure consistency in subject characteristics, we employ a graph contrastive learning strategy that aligns global and local feature representations. Results: Experimental results on the ABIDE-I dataset demonstrate that our model achieves an accuracy of 85.71%, outperforming most existing methods and confirming its effectiveness. Implications: The proposed model demonstrates superior performance in ASD classification, highlighting the potential of multi-atlas and multi-view learning for improving diagnostic precision and supporting early intervention strategies.

## 1. Introduction

Autism spectrum disorder (ASD) is a neurodevelopmental disorder characterized by social communication disorders, interest limitations, and repetitive behaviors. According to the latest estimates, one in every 150 children has ASD, and the global prevalence continues to rise [1]. The heterogeneity of ASD, along with its early onset and persistent impact, poses significant challenges for diagnosis and intervention. Early identification and intervention are of vital importance because the nervous system shows a high degree of plasticity in early childhood. Timely behavioral therapy, language training, and social skills development have been proven to alleviate symptoms, promote cognitive development, and improve long-term outcomes.

Resting-state functional magnetic resonance imaging (rs-fMRI) offers a non-invasive approach to studying the functional organization of the brain, providing crucial insights into the altered connection patterns that play a key role in diagnosing ASD [2]. By capturing spontaneous brain activity at rest, rs-fMRI enables researchers to identify subtle differences in neural networks that typically indicate autism-related abnormalities, thereby supporting more accurate and early diagnostic evaluations. In addition to its diagnostic value, it is equally important to consider the clinical perspectives associated with biomedical applications. From a safety monitoring standpoint, non-invasive imaging techniques such as rs-fMRI minimize potential risks compared with more invasive diagnostic approaches, thereby ensuring patient safety—a critical factor in clinical settings, especially for pediatric populations. Furthermore, understanding the interaction between imaging signals and biological tissues is essential for interpreting the resulting data accurately. The blood-oxygen-level-dependent (BOLD) signals captured in rs-fMRI, for instance, reflect complex neurovascular coupling mechanisms, which can vary across individuals and developmental stages. Accounting for these physiological interactions not only enhances diagnostic reliability but also helps bridge the gap between neuroimaging biomarkers and clinical decision-making in ASD detection.

Recently, there have been many applications that utilize resting-state functional magnetic resonance imaging data to support ASD classification tasks [3,4]. Almuqhim et al. proposed a model called the ASD-SAENet model and designed and implemented a sparse autoencoder to optimize the extraction of classification features [5]. Kan et al. proposed a low-level functional module based on self-supervised soft clustering and orthogonal clustering readout operations to determine the similar behaviors between ROI groups [6]. Wang et al. proposed a model called RGTNet. Specifically, a graph encoder was designed to extract time-dependent features with remote dependencies and a new graph sparse fitting weighted aggregation method was employed to alleviate the problem of dimensionality explosion [7]. However, most existing methods rely on a single brain map to define regions, which may limit the generalization and robustness of learning representations. To address such issues, Wen et al. proposed a multi-view convolutional neural network based on prior brain structure learning, which combines graph structure learning with multi-task graph embedding learning to enhance classification performance and identify potential functional subnetworks [8]. Zhu et al. developed a novel multi-view GNN for multimodal brain networks. They treated each modal as a view of the brain network and used contrastive learning for multi-modal fusion [9]. Song et al. used a multi-view attention fusion module to enhance the spectral convolutional network to extract useful information [10]. Although they all extract features from data based on different views, the performance of these models is poor, which is due to the inherent heterogeneity of the ABIDE dataset, while population graphs have a natural advantage in handling multi-site data. In the population graph, edge weights can be determined based on the phenotypic characteristics of the subjects (such as collection location, age, and gender), further weakening the impact of differences in different sites, age groups, and gender on the data.

In terms of the application of population graphs, Cao et al. constructed a deep ASD diagnosis framework based on 16-layer GCN. Integrating ResNet cells and DropEdge policies into this framework avoids problems such as vanishing gradients, overfitting, and oversmoothing [11]. Tian et al. proposed an extensible hierarchical graph convolutional network. Firstly, they constructed GCN based on brain regions of interest to extract structural and functional connection features between different ROIs, and combined them with scale information to build a population-based GCN model [12]. Although these methods can alleviate the impact of data heterogeneity, they lack feature representations of different views.

The research deficiencies of the specific existing methods are shown in Table 1. To address these deficiencies and integrate the advantages of these methods, we propose a multi-atlas guided multi-view contrast learning (MAMVCL) for ASD classification. Our main contributions are as follows:

(1) Our method constructs functional connectivity matrices based on three distinct brain atlases, capturing diverse perspectives of brain functional organization.
(2) The interaction between the brain regions of the subjects was conducted through the Target-aware attention aggregator layer to obtain the local features of all subjects. By constructing population graphs among different subjects and performing graph convolution operations, the global features of all subjects are obtained for message passing among similar subjects to obtain global features.
(3) We adopt graph contrastive learning to align global and local feature representations, and use consistency loss to constrain different brain atlas feature representations of the same encoder.
(4) Extensive experiments on the Autism Brain Imaging Data Exchange (ABIDE-I) dataset demonstrate that our model achieves an accuracy of 85.71%, outperforming existing state-of-the-art approaches.

**Table 1:** The flaws of the existing methods.

Category	Defect
Single-view model	The model limits the generalization and robustness of learning representations
Multi-view model	The performance of the model is poor and the heterogeneity of the data has not been alleviated
Population graph model	The model lacks feature representations of different views

The structure of this paper is organized as follows: Section 2 details the proposed model, including its methodologies and the loss functions employed. Section 3 presents the experimental setup, featuring comparative experiments, ablation studies, and more. Section 4 discusses key aspects such as hyperparameters, interpretable analysis, and more. Section 5 concludes the paper and outlines directions for future research.

## 2. Materials and Methods

**Figure 1:**
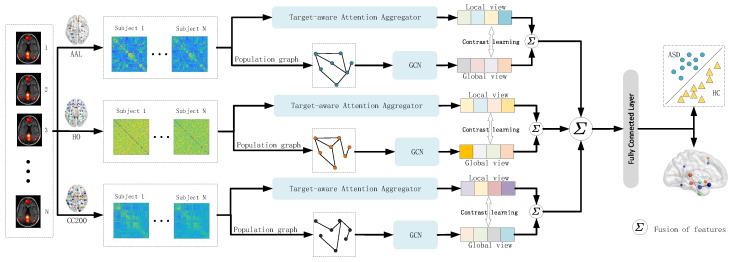
Overview of the proposed graph learning framework based on multi-atlas integration. This model integrates the feature representations derived from three different brain atlas views. Within each atlas framework, both the imaging features extracted from the brain atlas and the phenotypic information of the subjects are utilized to construct their respective population graphs. A GCN is then applied to these graphs to capture global representations. Meanwhile, subject-specific local features are extracted through a Target-aware attention aggregator layer. Finally, graph contrastive learning is performed between the global and local features to enhance representation robustness and discriminative ability.

**Figure 2:**
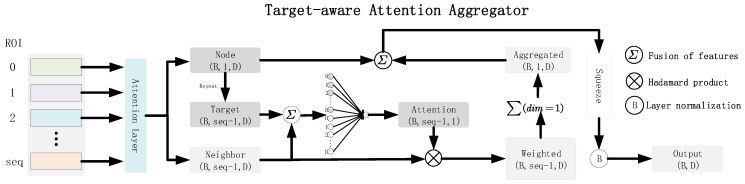
Structure of the Target-aware attention aggregator layer. This figure illustrates the detailed architecture of the Target-aware attention aggregator layer, highlighting how the self-attention mechanism interacts with various regions of interest (ROIs). Through this mechanism, the model adaptively emphasizes task-relevant regions while capturing their relational dependencies, thereby enhancing the extraction of subject-specific local features.

The overall framework of the model proposed in this paper is shown in Figure 1. The model integrates the feature representations of three different brain atlas visuals. In each brain atlas framework, the imaging features extracted from the brain atlas and the phenotypic features of the subjects are used to construct their respective population graph. Then, on the constructed population graph, the graph convolution operation is applied to obtain the global features of the subjects. Meanwhile, the Target-aware attention aggregator layer (Figure 2) was used to interact between the brain regions of the subjects and extract local features. Then, graph contrastive learning is conducted on the global and local features to ensure the consistency of the feature representation of the subjects [13]. Finally, the improved features are applied to the classification task.

### 2.1. Target-Aware Attention Aggregator

The multi-head self-attention (MSA) mechanism is designed to capture semantic correlations between input tokens [14]. We added the MSA mechanism at the beginning of the Target-aware attention aggregator module.

To effectively capture interaction patterns between brain regions, we have designed a Node-Centric Attention Aggregation (NCAA) module. Given the brain network representation $X^{msa}\in R^{B\times seq\times D}$, where *B* denotes the batch size, seq represents the number of brain regions, and *D* indicates the feature dimension, the NCAA module centers on each brain region to aggregate information from the remaining regions, thereby enhancing node representations.

Specifically, for the *i*-th brain region, we begin by concatenating its feature vector $h_{i}$ with the feature vectors of its neighboring nodes $\left\{h_{j}|j\neq i\right\}$. This concatenated input is then fed into an attention mapping function $Attn_{i}\left(\cdot \right)$, which generates the attention weight distribution for the neighboring nodes.

(1) $$\;\alpha_{ij}=\mathrm{Softmax}\;\left({\mathrm{Attn}}_{i}\;\left([\;h_{i}\|h_{j}\;]\right)\right),\;j\neq i$$

Subsequently, we apply these weights to perform a weighted aggregation of the neighbor features, which is then combined with the center node’s own features to derive the updated node representation.

(2) $$\;{\tilde{h}}_{i}=h_{i}+\sum_{j\neq i}\alpha_{ij}h_{j}$$

Ultimately, the enhanced representations of all nodes are concatenated to form an updated brain network representation.

(3) $$\;X^{te}=\left[\;{\tilde{h}}_{1},{\tilde{h}}_{2},\ldots ,\;{\tilde{h}}_{seq}\;\right]\in R^{B\times seq\times d}$$

In this manner, the NCAA module dynamically models the dependencies of each brain region with the rest of the brain, centering on individual regions to highlight task-relevant interaction patterns while suppressing irrelevant or noisy connections.

### 2.2. Population Graph Construction

#### 2.2.1. The Nodes of the Population Graph

We calculate the average time series of each brain region in the rs-fMRI image data and obtain the functional connectivity matrix $X^{fc}$ using the Pearson correlation coefficient. The elements of the upper triangular matrix are chosen as the node initial features $X^{ut}$ of the population graph. The total number of subjects across all sites is *N*.

#### 2.2.2. The Edges of the Population Graph

In a population graph, edges signify the similarity between pairs of samples. To form these edges, we combine imaging data with phenotypic information, including site and age, within a scoring mechanism to refine the population graph. For the retained edges, the cosine similarity between the phenotypic features of the paired subjects is computed to establish the final edge weights [15].

To construct a sparse population graph with enhanced connections among similar subjects, we implement a scoring mechanism to filter edges, yielding a binarized matrix *F*, calculated as detailed in Equation (4):

(4) $$\;F_{ij}=\left\{\begin{array}{c} 0,\;if\;A_{ij}<\theta_{1}\\ 1,\;if\;A_{ij}\geq \theta_{1} \end{array}\right.$$

where $A\in R^{N\times N}$ represents the score matrix of each edge in the fully connected population graph, and $\theta_{1}$ is a hyperparameter that adjusts the sparsity of the population graph. The matrix A is calculated as shown in Equation (5)

(5) $$\;A_{ij}=X_{ij}^{ut}\cdot \sum_{m=1}^{N_{m}}\varphi \left(P_{im},P_{jm}\right)$$

where $X_{ij}^{ut}$ represents the similarity of the imaging data between the *i*-th and *j*-th subjects. φ(·) represents the function for calculating the similarity between the feature tables of two subjects. $N_{m}$ represents the number of phenotypic data, and $P_{im}$ indicates the *m*-th phenotypic feature of the *i*-th sample.

For categorical information such as site information, the calculation method of φ(·) is shown in Equation (6):

(6) $$\;\varphi \left(P_{im},P_{jm}\right)=\left\{\begin{array}{cc} 0, & \;if\;P_{im}\neq P_{jm}\\ 1, & \;if\;P_{im}=P_{jm} \end{array}.\right.$$

For quantifiable data such as age, the calculation method of φ(·) is shown in Equation (7):

(7) $$\;\varphi \left(P_{im},P_{jm}\right)=\left\{\begin{array}{cc} 1, & \;if|P_{im}-P_{jm}|<\theta_{2}\\ 0, & \;if|P_{im}-P_{jm}|\geq \theta_{2} \end{array}\right.$$

where $\theta_{2}$ is a hyperparameter, which is a manually adjusted threshold.

To uncover hidden relationships among subject characteristics, we utilize an attention mechanism to augment the dimensionality of their phenotypic features *P*. The cosine similarity between these enhanced features is then computed to establish the edge weights. Leveraging the previously derived binarized matrix *F*, we filter the edges of the population graph. The weighted adjacency matrix *W* of the population graph is calculated as follows:

(8) $$\;W_{ij}=\left\{\begin{array}{cc} \frac{Cos\left(P_{i},P_{j}\right)+1}{2}, & \;if\;F_{ij}=1\\ 0, & \;if\;F_{ij}=0 \end{array}\right.$$

where Cos(·) represents the cosine similarity calculation.

It is worth noting that similar principles of feature enhancement and signal integration are widely explored in other biomedical domains, particularly at the hardware and interface level. For example, the design of electronic interfaces for single-photon avalanche diodes (SPADs) has been shown to significantly improve the sensitivity and temporal resolution of biomedical imaging systems by optimizing signal acquisition and noise suppression [16,17]. Such interface-level innovations share conceptual similarities with our approach: both aim to maximize the extraction of meaningful information from complex, heterogeneous data sources — whether through physical electronic circuitry or through graph-based modeling of subject relationships. Integrating these perspectives provides a broader context for understanding how the proposed population graph serves as an “interface” between raw imaging data, phenotypic characteristics, and downstream analytical tasks.

### 2.3. Graph Convolutional Network

The model proposed in this paper utilizes a multi-layer GCN, with its hierarchical propagation rule defined as follows:

(9) $$\;H^{\left(l+1\right)}=\mathrm{Relu}\left({\tilde{D}}^{-1/2}\tilde{A}{\tilde{D}}^{-1/2}H^{\left(l\right)}W^{\left(l\right)}\right)$$

where $\tilde{A}=A+I_{N}$ is the adjacency matrix of an undirected graph, augmented with the identity matrix $I_{N}$ to include self-loops, $\tilde{D}$ is the degree matrix of $\tilde{A}$, $W^{\left(l\right)}$ is the trainable weight matrix for layer *l*, and $H^{\left(l\right)}$ is the activation matrix at layer *l*, with $H^{\left(0\right)}=X^{ut}$ serving as the initial input feature matrix.

Prior to GCN convolution, a DropEdge strategy is implemented, which randomly removes a subset of edges from the input graph during each training iteration [18]. However, no edges are removed during testing to preserve the graph’s integrity. This approach helps address overfitting. Additionally, residual connections are introduced in each convolutional layer to retain complete feature information, enhancing the model’s robustness [19].

### 2.4. Perturbation-Driven Explainable Framework

The core idea of this model is to use the complementarity of different brain atlases in brain region division to improve the classification performance. However, due to the complexity of multi-atlas models and other factors, the use of a model based on a single brain atlas for analysis is more convincing in terms of medical interpretation. First, we trained the model based on a single brain atlas to obtain the corresponding best classification accuracy $Acc^{*}$, which was used as a reference benchmark for subsequent interpretability experiments

To quantitatively assess the importance of various brain regions in the classification task, we developed perturbation-based experimental methods. Specifically, for a subject under a given brain atlas, we consider two inputs: the functional connectivity matrix $S\in R^{M\times M}$ and its upper triangular feature $U\in R^{d}$, where *M* represents the number of brain regions defined by the atlas, and d=M×(M−1)/2 denotes the dimension of the upper triangular feature. Then we set the index of the brain region with experiment as *i*, and we removed the features related to *i* on *S* and *U*, respectively:

(10) $$\;\tilde{S}=Mask_{1}\left(S\right),\;\tilde{U}=Mask_{2}\left(U\right)$$

Here, the mask functions $Mask_{1}\left(\cdot \right)$ and $Mask_{2}\left(\cdot \right)$ denote zeroing or removing the features associated with index *i* in *S* and *U*, respectively. Subsequently, the perturbed features $\tilde{S}$ and $\tilde{U}$ are input into the trained model to obtain the corresponding classification accuracy $\tilde{Acc}$.

By comparing $\tilde{Acc}$ with the best benchmark accuracy $Acc^{*}$, we defined the importance index of brain regions as:

(11) $$\;\Delta Acc=Acc^{*}-\tilde{Acc}$$

Among them, a larger ΔAcc indicates that the masked brain region *i* is more important in the classification task. This analysis can be combined with different brain atlas perspectives to reveal the potential biological significance of brain regions in disease classification.

### 2.5. Loss Function

#### 2.5.1. Muti-Altas Consistency Loss Function

As our model integrates multiple brain atlases, which represent distinct perspectives of the same brain network, they should exhibit consistency in the final analysis. To achieve this, we introduce consistency constraints to optimize the similarity across different views [20], regularized as follows:

(12) $$\;L_{\mathrm{vc}}=-\sum_{(i,j)\in V,i\neq j}\log\sigma \;\left(G_{i}{\left(G_{j}\right)}^{⊤}+T_{i}{\left(T_{j}\right)}^{⊤}\right)$$

where $G_{i}$ represents the membership vector of the *i*-th view following graph convolution learning, $T_{i}$ denotes the membership vector of the *i*-th view after Target-aware attention aggregator layer learning, and *V* constitutes the set of distinct views within our model. Incorporating this loss function encourages convergence between the model outputs for the *i*-th and *j*-th views.

#### 2.5.2. Muti-View Contrastive Loss Function

To enhance the consistency of representations across different perspectives, we have designed a contrastive loss function [13]. Let the representations output by two distinct models or branches be $z^{lv}$ and $z^{gv}$. These are first mapped to a shared latent space using a common non-linear projection head:

(13) $$\;\phi^{lv}=\mathrm{Proj}\left(z^{lv}\right),\;\phi^{gv}=\mathrm{Proj}\left(z^{gv}\right)$$

The projection function consists of two linear transformations combined with a non-linear activation (ELU), employing Xavier initialization to ensure numerical stability.

Subsequently, we define the similarity between the two representations as follows:

(14) $$\;\mathrm{sim}\left(\phi_{i},\phi_{j}\right)=\exp\;\left(∥\phi_{i}∥\cdot ∥\phi_{j}∥\cdot \tau \;\phi_{i}^{⊤}\phi_{j}\right)$$

where τ serves as a temperature parameter, used to adjust the smoothness of the distribution. Based on this similarity, we construct normalized similarity matrices from $\phi^{lv}$ to $\phi^{gv}$ and from $\phi^{gv}$ to $\phi^{lv}$. These are then utilized, along with a given positive sample mask $M^{p}$, to compute the contrastive loss:

(15) $$\;L_{lv}=-\frac{1}{N}\sum_{i=1}^{N}\log\frac{\sum_{j}\mathrm{sim}\;\left(\phi_{i}^{\mathrm{lv}},\;\phi_{j}^{\mathrm{gv}}\right)\cdot M_{ij}^{p}}{\sum_{j}\mathrm{sim}\;\left(\phi_{i}^{\mathrm{lv}},\;\phi_{j}^{\mathrm{gv}}\right)}$$

(16) $$\;L_{gv}=-\frac{1}{N}\sum_{i=1}^{N}\log\frac{\sum_{j}\mathrm{sim}\;\left(\phi_{i}^{\mathrm{gv}},\;\phi_{j}^{\mathrm{lv}}\right)\cdot M_{ij}^{p}}{\sum_{j}\mathrm{sim}\;\left(\phi_{i}^{\mathrm{gv}},\;\phi_{j}^{\mathrm{lv}}\right)}$$

The final total loss is a weighted combination of the two:

(17) $$\;L_{cl}=\gamma L_{lv}+\left(1-\gamma \right)L_{gv}$$

where γ acts as a balancing coefficient.

This loss function explicitly aligns the representation spaces of the two distinct model outputs, enhancing the consistency and discriminability of representations across perspectives.

#### 2.5.3. Cross-Entropy Loss Function

This paper focuses on a binary classification task, utilizing the cross-entropy loss as the primary predictive loss function [21], defined as follows:

(18) $$\;L_{ce}=-\frac{1}{N}\sum_{n=1}^{N}\left[y_{n}\log\left({\hat{y}}_{n}\right)+\left(1-y_{n}\right)\log\left(1-{\hat{y}}_{n}\right)\right]$$

Here, $y_{n}\in \left\{0,1\right\}\;$ denotes the true label of subject *n*, and ${\hat{y}}_{n}\in \left(0,1\right)$ represents the predicted probability of the positive class.

Combining the above components, the final loss function is:

(19) $$\;L=L_{ce}+\alpha L_{vc}+\beta L_{cl}$$

Here, α and β are hyperparameters that balance the contributions of each loss term.

## 3. Results

### 3.1. Data Acquisition and Pre-Processing

The experimental data for this study were derived from rs-fMRI data within the initial phase of the ABIDE-I database, a publicly accessible multi-site repository containing data from 1112 individuals across 17 sites. We employed the Configurable Pipeline for the Analysis of Connectomes (CPAC) [22] for image preprocessing, encompassing slice-timing correction, motion correction, skull stripping, and voxel intensity normalization. This study generated 875 high-quality MRI images, comprising 405 individuals with ASD and 470 typically developing controls (TC). Detailed demographic data are provided in the accompanying Table 2.

Subsequently, using the Harvard–Oxford (HO) [23], Automated Anatomical Labeling (AAL) [24], and Craddock 200 (CC200) brain atlases [25], respectively, the preprocessed data were mapped onto various regional levels. The Pearson correlation coefficient was then computed between the average time series of all regions of interest (ROIs) across these different brain atlases to construct the functional connectivity matrices of brain networks from multiple perspectives.

### 3.2. Experimental Setup

#### 3.2.1. Experimental Parameter

The edge filtering threshold is set to $\theta_{1}=0.61$, the quantifiable data scoring mechanism parameter to $\theta_{2}=2$, and the loss function parameters to α=0.1 and β=0.01. For a fair comparison, the source codes of all competing methods were run in the same environment, with hyperparameters configured according to the optimal settings recommended in their respective papers. Detailed equipment and model parameters are presented in the Table 3.

#### 3.2.2. Performance Evaluation

We implemented a 10-fold cross-validation approach to evaluate the efficacy of our algorithm. For performance assessment, we adopted accuracy (*ACC*), precision (*PRE*), recall (*RECALL*), F1 score (*F1*), and area under the curve (*AUC*) as primary metrics. The calculation methods for these performance metrics are detailed as follows:

(20) $$\;ACC=\frac{TP+TN}{TP+TN+FP+FN}$$

(21) $$\;PRE=\frac{TP}{TP+FP}$$

(22) $$\;RECALL=\frac{TP}{TP+FN}$$

(23) $$\;F1=\frac{2\times PRE\times RECALL}{PRE+RECALL}$$

where TP, TN, FP, and FN represent true positives, true negatives, false positives, and false negatives, respectively.

### 3.3. Competing Methods

Among the comparative methods, we chose two prominent baseline techniques: Support Vector Machine (SVM) and Random Forest (RF). Additionally, we assessed several cutting-edge deep learning networks, including ASD-DiagNet, ASD-SADnet, BrainGNN, DeepGCN, BNT, FBNetGen, MVS-GCN, GCN-MDD, TP-MIDA, deepManReg, DG-DMSGCN, RGTNet, GBT, and CcSi-MHAHGEL. A concise overview of these 16 methods is provided below.

(1) Random Forest and SVM: The upper triangular vector of the functional connectivity matrix serves as the feature set, with classifiers implemented using Random Forest and Support Vector Machine techniques.
(2) ASD-DiagNet [26]: The ASD-DiagNet model employs a joint learning approach, integrating autoencoders with single-layer perceptrons to improve the quality of extracted features and enhance classification performance.
(3) ASD-SAENet [5]: The ASD-SAENet model is designed to classify patients with ASD from typical control subjects using fMRI data, integrating a sparse autoencoder (SAE) to optimize feature extraction, which is then fed into a deep neural network (DNN) to enable enhanced classification of ASD-prone fMRI brain scans.
(4) BrainGNN [27]: The BrainGNN model based on graph neural networks facilitates ASD prediction by utilizing two types of Brain Functional Networks (BFNs) as adjacency matrices.
(5) Deep-GCN [11]: The Deep-GCN model develops a comprehensive ASD diagnostic framework utilizing a 16-layer population GCN.
(6) Brain network transformer (BNT) [6]: The BNT model integrates a self-attention mechanism and introduces an orthogonal clustering readout operator, utilizing self-supervised soft clustering and orthogonal projection methods.
(7) FBNetGen [28]: The FBNetGen model focuses on the learnable generation of brain networks while exploring the interpretability of these generated networks for downstream applications.
(8) MVS-GCN [8]: The MVS-GCN model employs threshold-based measures to partition an adjacency matrix into three matrices with varying sparsity levels, thereby improving the comprehensiveness of the derived features.
(9) GCN-MDD [29]: Integrates the k-Nearest Neighbors (kNN) algorithm to construct graphs and employs a GCN model for disease prediction.
(10) TP-MIDA [30]: The TP-MIDA model leverages the Tangent Pearson embedding method to extract features and applies domain adaptation techniques to reduce the site-specific dependence of functional connectivity features.
(11) deepManReg [31]: The deepManReg model employs multiple deep neural networks tailored to different modalities, jointly training them to align multimodal features into a shared latent space, and subsequently utilizes cross-modal manifolds to regularize the classification network, enhancing phenotype prediction accuracy.
(12) DG-DMSGCN [32]: The DG-DMSGCN model incorporates a sliding window dual-GCN to extract features while capturing the spatiotemporal characteristics of fMRI data across varying sequence lengths. Subsequently, a novel dynamic multi-site GCN is employed to aggregate features derived from multiple medical sites.
(13) RGTNet [7]: The RGTNet model develops a graph encoder to extract time-dependent features with long-range dependencies and introduces a novel sparse graph fitting weighted clustering method to mitigate the dimensionality explosion challenge.
(14) GBT [33]: The GBT model introduces a Transformer module that selectively eliminates small singular values from the attention weight matrix to capture the most relevant graph representations. Additionally, it incorporates a geometry-oriented representation learning module, which enforces low-order intra-class compactness and high-order inter-class diversity constraints on the learned representations to enhance their discriminability.
(15) CcSi-MHAHGEL [34]: The CcSi-MHAHGEL model introduces a Class-Consistency and Site-Independence Multiview Hyperedge-Aware HyperGraph Embedding Learning framework, designed to integrate Functional Connectivity Networks (FCNs) constructed from multiple brain atlases within a multi-site fMRI study.

### 3.4. Results of Comparison Methods

Table 4 presents the quantitative results for each key performance metric: *ACC*, *Pre*, *Recall*, *F1*, and *AUC*. All values are reported as mean ± standard deviation (SD), providing a robust measure of the model’s stability across 10 cross-validation folds.

The proposed MAMVCL model demonstrated the highest performance across all metrics, achieving an *ACC* of 85.71%. This represents a substantial improvement over baseline methods, such as Random Forest and SVM, underscoring the limitations of traditional machine learning approaches in effectively handling the inherent complexity and heterogeneity of neuroimaging data. Compared to state-of-the-art deep learning models, MAMVCL outperformed graph-based methods including BrainGNN, Deep-GCN, and CcSi-MHAHGEL, as well as attention- and transformer-oriented models such as BNT, GBT, and RGTNet. These results indicate enhanced discriminability and robustness of MAMVCL, particularly against class imbalance.

The superior performance of MAMVCL highlights its ability to effectively integrate multi-atlas features, extract the global features of the subjects by using the convolution of the population graph, extract the local features of the subjects by using the Target-aware attention aggregator layer, and constrain the consistency of the feature representation of the subjects by using graph contrastive learning. This combination enables the model to mitigate the impact of noise in the data, address the variability of specific sites, and capture complex brain connection patterns, which are crucial for accurate ASD classification.

### 3.5. Ablation Experiment

To assess the effectiveness of the Target-aware attention aggregator (TAA) and graph contrast learning (GCL) modules within our proposed model, we conducted experiments on four distinct variants. These variants are defined as follows:

(1) Variant I: Employs only the GCN module utilizing population graphs.
(2) Variant II: Relies solely on the Target-aware attention aggregator module.
(3) Variant III: Combines the Target-aware attention aggregator module with the GCN module for population graphs, but omits graph contrast learning to align the outputs of the same subjects.

The experimental results for these variants are presented in Table 5. The data indicate that our full model surpasses the performance of all variants, demonstrating that the integration of the TAA and GCL modules significantly enhances the model’s effectiveness. Specifically, the TAA module, grounded in individual brain networks, provides a localized perspective, while the GCN module, applied to population graphs, captures implicit relationships between subjects, offering a global viewpoint. Graph contrast learning further refines the model by aligning the output features of these two modules, ensuring a comprehensive and cohesive final output.

To quantitatively evaluate the superiority of the proposed model and the contributions of each component, we conducted a paired t-test on the classification accuracy obtained from 30 repeated cross-validation runs. Variant III (integrating TAA and GCN) performed significantly better than Variant I (containing only TAA) (*p* = 4.1710^−30^) and Variant II (containing only GCN) (*p* = 3.8710^−19^). Furthermore, the complete MAMVCL model containing the GCL module has a much higher accuracy rate than Variant III (*p* = 5.4210^−13^). These results confirm that each component makes a significant contribution to the overall performance, and the proposed model has higher classification accuracy statistically compared with its dissolved variants.

## 4. Discussion

### 4.1. Phenotypic Information Analysis

Table 6 summarizes the impact of various phenotypic feature combinations on model performance. Among individual features, site (S) consistently delivered the highest results, achieving an *ACC* of 84.28% and an *AUC* of 91.76%, markedly outperforming age (A) and gender (G) [35]. This indicates that site information plays a predominant role in model classification. When examining feature combinations, the S + A pairing yielded the best performance (*ACC* = 85.71%, F1 = 86.84%, *AUC* = 92.93%), suggesting a complementary effect between site and age that bolsters the model’s discriminative capability. In contrast, the S + G combination resulted in reduced performance, while the A + G pair exhibited the weakest outcome, highlighting the limited discriminative power of gender, either alone or in combination. Incorporating additional phenotypic traits, such as eye status (E), handedness (H), and IQ score (FVP) [36], did not lead to further improvements, with performance stabilizing between 80% and 82%, falling short of the optimal S + A combination. This implies that adding more phenotypic data may introduce redundancy or noise, thereby diminishing overall effectiveness. Collectively, these findings emphasize that site is the most informative feature, with age providing complementary benefits when paired with site. The contributions of other features appear minimal or even detrimental.

Therefore, the S + A combination is recommended as the most effective set of phenotypic features for constructing a population graph model. This is also in line with the research results of slopen [37].

### 4.2. Interpretability Analysis

**Figure 3:**
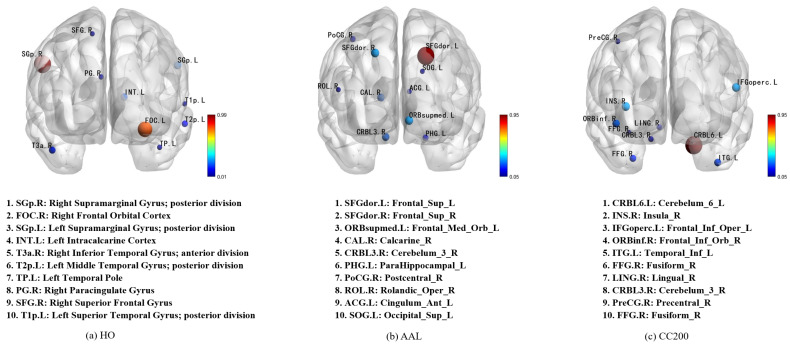
Visualization of the most important brain regions across different atlases. The ten most important brain regions identified from the HO, AAL, and CC200 atlases are visualized, with node size indicating their contribution strength to the model. The full names and abbreviations of these regions are listed in descending order of importance, providing an interpretable view of feature relevance under different parcellation schemes.

**Figure 4:**
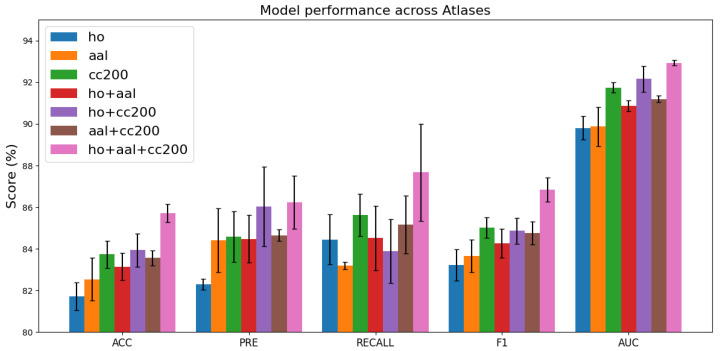
Comparison of model performance across different brain atlas combinations. Variations in performance metrics under different atlas configurations illustrate how parcellation choice influences classification accuracy and predictive effectiveness.

To clarify the proposed model, we employed perturbation experiments to identify the most distinctive brain regions. These experiments were conducted on three different brain atlases used in the research (Figure 3). Since the CC200 map was generated in our analysis through monomeric BOLD time series clustering and lacked a predefined label for each ROI, we aligned its 200 ROis with the AAL brain map based on their spatial positions to enhance our understanding of the potential classification mechanism. The ten most important features in each mind map are identified and highlighted. The interpretable analyses of the HO, AAL, and CC200 brain atlases are respectively shown in Figure 4.

By observing the first and second images in Figure 4, it was found that the region most relevant to ASD diagnosis is located at Right Supramarginal Gyrus. This finding was supported by Wada et al., who demonstrated that this region is associated with the maintenance of emotion recognition ability. The ability to recognize emotions is closely related to ASD symptoms [38]. In the second images, the two most important features, Frontal_Sup_L and Frontal_Sup_R, correspond to the dorsolateral frontal gyrus, a region medically considered to play a key role in language understanding [39]. Combining the first and third figures, we also found that the area around Cerebelum_6_L was identified as the region most relevant to ASD classification. The researchers confirmed that this link was previously shown to have a significant relationship with ASD pathology [40,41,42].

In addition, our population-level perturbation analysis shows conceptual parallels with finite element modeling (FEM)-based approaches for population-specific brain modeling, such as the study by [43]. Both strategies aim to capture how localized structural or functional perturbations propagate through the broader system to affect global behavior. In FEM-based population models, the spatial distribution of stress or strain fields is used to infer regions most responsible for functional deviations. Similarly, our multi-atlas perturbation framework identifies brain regions whose alterations most significantly influence ASD classification outcomes. This analogy underscores that both methods—despite differing in implementation—share a common objective: revealing region-specific contributions to system-level properties in a non-invasive and interpretable manner. Integrating such perspectives enriches the interpretability of our model and highlights its potential relevance for broader biomedical modeling applications.

### 4.3. Different Brain Atlas Combinations on the Model

Figure 4 illustrates the classification performance across various brain atlas configurations. Models trained on a single atlas, such as HO, AAL, or CC200, exhibit *ACC* values ranging from 81% to 83%. In contrast, integrating multiple atlases consistently enhances performance, with the combination of all three atlases (HO + AAL + CC200) yielding the best results, achieving an *ACC* of approximately 85.7%. These findings indicate that the complementary information from diverse brain regions strengthens the robustness of feature representations and enhances the model’s generalization capability. Moreover, the low standard deviations observed in most cases reflect stable performance, while the three-atlas configuration delivers the highest average performance with relatively manageable variability.

### 4.4. Hyperparametric Analysis

#### 4.4.1. The Influence of the Loss Function

**Figure 5:**
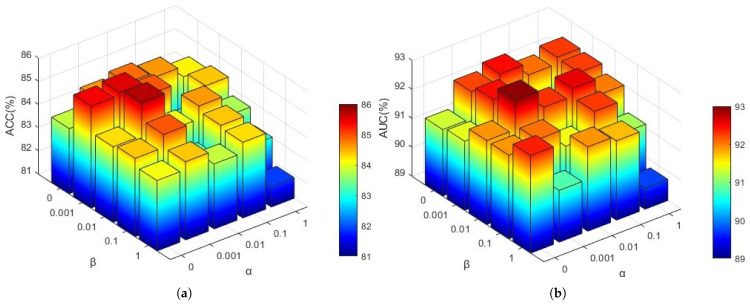
Impact of hyperparameter settings on model performance. Comparison of performance metrics under different hyperparameter configurations reveals the model’s sensitivity to key parameters and highlights optimal settings for improved predictive accuracy. (**a**) *ACC*; (**b**) *AUC*.

To investigate the influence of hyperparameters in Equation (19), we analyzed the effects of view consistency loss (α) and contrast loss (β) on model performance. Employing a grid search approach, we evaluated various combinations of these coefficients with values [0, 0.001, 0.01, 0.1, 1]. The resulting *ACC* and *AUC* values are presented in Figure 5.

As illustrated in Figure 5, the optimal *ACC* and *AUC* values are achieved when α=0.001 and β=0.01. This suggests that an excessively small α value may prevent the outputs of different brain atlases for the same subject from aligning closely, whereas an overly large α could alter the effective feature values used for classification across different views. Similarly, a β value that is too small may lead to significant disparities between the global and local features of the same subject, while an excessively large β might enforce excessive consistency between global and local feature regions.

In this study, hyperparameter optimisation was conducted using a grid search approach, which exhaustively explores the parameter space and is commonly used in machine learning tasks due to its simplicity and interpretability. Alternative approaches such as Taguchi design or ANOVA-based optimisation could potentially improve efficiency by reducing the search space and considering factor interactions. However, since our model involves a relatively small set of hyperparameters, grid search was sufficient and computationally manageable.

#### 4.4.2. The Influence of Constructing the Edges of the Population Graph

The hyperparameters $\theta_{1}$ and $\theta_{2}$ play a critical role in shaping the structure of the population graph by determining how edges are formed between subjects. Specifically, $\theta_{1}$ controls the similarity threshold for connecting nodes based on their feature representations, while $\theta_{2}$ regulates the influence of age proximity during the construction of graph edges. To determine an appropriate range for $\theta_{1}$, we first conducted a preliminary sensitivity analysis by varying its value within the range of 0.3 to 0.7. The results revealed that the model exhibited consistently better classification performance when $\theta_{1}$ was set between 0.60 and 0.65. Based on this observation, we subsequently adopted this narrower interval as the search space for grid-based hyperparameter tuning, allowing a more precise exploration of the optimal threshold.

**Figure 6:**
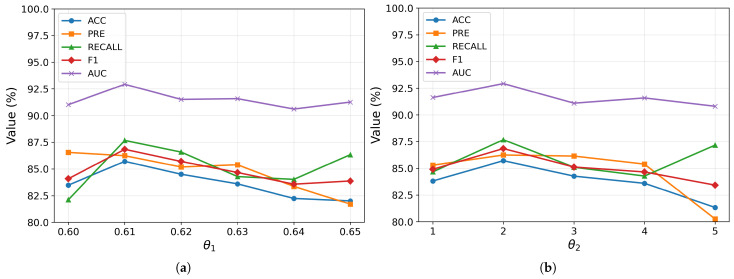
Impact of $\theta_{1}$ (**a**) and $\theta_{2}$ (**b**) on model performance. Variation of $\theta_{1}$ affects the construction of the population graph and alters message passing dynamics, leading to changes in classification performance. Variation of $\theta_{2}$ affects message propagation based on age similarity, thereby influencing classification outcomes.

As shown in Figure 6, the model achieves optimal classification performance when $\theta_{1}=0.61$ and $\theta_{2}=2$, indicating a delicate balance between connectivity and noise. If $\theta_{1}$ is set too low, the resulting graph becomes overly dense, introducing numerous spurious edges that degrade the discriminative capacity of the learned representations. Conversely, an excessively high $\theta_{1}$ leads to a sparse graph, which restricts information flow among similar subjects during message passing. A similar trade-off is observed for $\theta_{2}$: smaller values hinder effective communication among nodes with similar age characteristics, while larger values introduce irrelevant age-related connections that may obscure the underlying patterns. These observations underscore the importance of jointly tuning $\theta_{1}$ and $\theta_{2}$ to achieve a graph topology that both preserves meaningful relationships and suppresses noise, ultimately enhancing the quality of representation learning.

#### 4.4.3. The Influence of Parameters in Graph Contrast Learning

**Figure 7:**
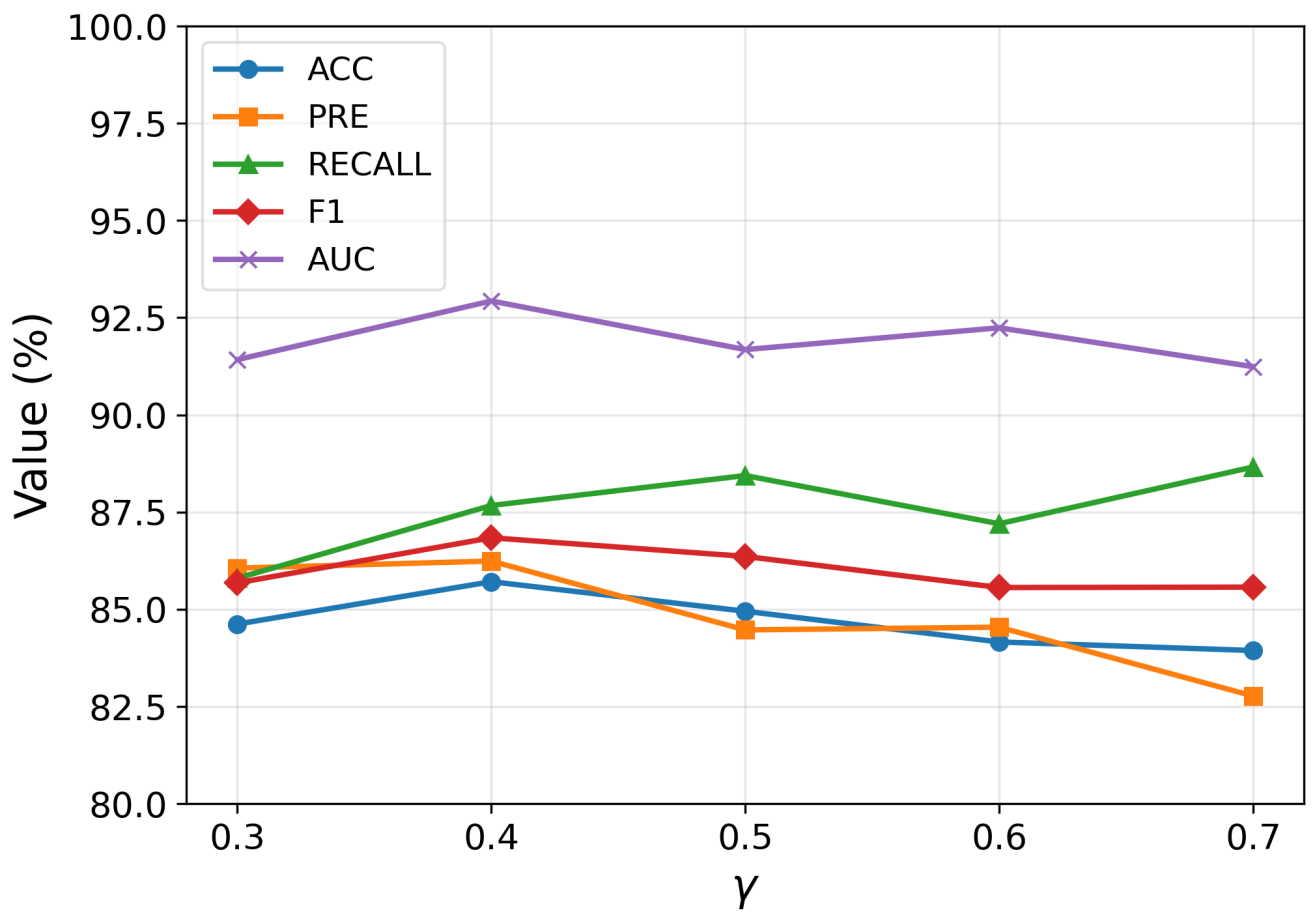
Impact of γ on representation alignment. The balancing coefficient γ regulates the alignment between local and global representations in the contrastive learning framework.

The hyperparameter γ directly affects the optimization objective in the contrastive learning stage by balancing the alignment between local and global representations. As depicted in Figure 7, the model exhibits its best performance when γ=0.4. This result suggests that an appropriate weighting of the two alignment objectives is essential for effective representation learning. When γ is too small, the contribution of local information is underemphasized, potentially leading to insufficient capture of fine-grained structural patterns. Conversely, an excessively large γ can overemphasize local alignment at the expense of global semantic consistency, resulting in suboptimal generalization. The optimal value reflects a scenario in which global context plays a more dominant role than local details, highlighting the significance of integrating broader structural information in the contrastive framework. This balance allows the model to learn robust, semantically meaningful representations that improve downstream classification performance.

#### 4.4.4. The Attention Layers in Target-Aware Attention Aggregator Layer

**Figure 8:**
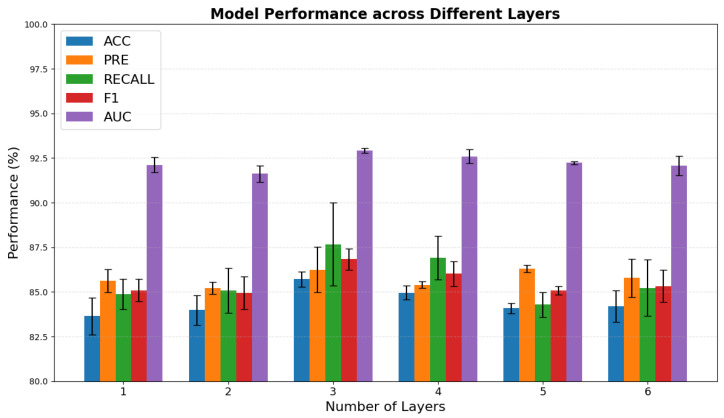
Impact of attention layer configurations on model performance. Different attention layer designs lead to variations in classification metrics, highlighting how attention mechanisms influence overall model effectiveness.

The results presented in Figure 8 illustrate the impact of varying the number of attention layers on the model’s performance. Notably, the configuration with three attention layers achieved the highest overall performance, including the best *ACC*, suggesting that this structure provides sufficient representational capacity without leading to overfitting. When the number of layers falls below three, the model exhibits clear signs of underfitting, failing to adequately capture local features. Conversely, increasing the number of layers beyond three results in a slight decline or fluctuation in performance, indicating that deeper attention stacking does not consistently enhance generalization and may introduce redundancy or noise. Therefore, these findings suggest that three attention layers strike an optimal balance between model complexity and generalization capability.

### 4.5. Limitations and Future Work

Although our model demonstrates good performance, there are still several limitations. First, the construction of the population graph requires knowledge of the entire dataset, which means that incorporating new subjects necessitates retraining the mode—a limitation that affects scalability in real-world scenarios. Second, the use of multiple atlases, while improving feature diversity, increases the computational cost and training complexity. Finally, the current framework relies solely on resting-state functional connectivity, and does not yet leverage complementary information from other imaging modalities such as structural MRI or diffusion imaging. To further evaluate the reliability of our framework, it is essential to consider perspectives inspired by stress analysis, particularly in terms of uncertainty management and feature robustness. In real-world scenarios, data are often affected by noise, variability, and acquisition inconsistencies, which may influence model performance. Our approach demonstrates stable classification results even under moderate perturbations, indicating a certain degree of robustness. Nevertheless, incorporating uncertainty-aware strategies—such as Bayesian modeling, dropout-based inference, or sensitivity analysis—could further enhance the model’s reliability.

Future research will aim to address these limitations in several ways. One direction is to explore incremental or fine-tuning strategies to enable efficient model adaptation to new samples without the need for full retraining. Specifically, this could involve transfer learning techniques to reuse and adapt pre-trained model weights for new data distributions, incremental learning approaches that progressively update the model as new samples become available, or online learning frameworks capable of continuously integrating streaming data. Such strategies would significantly enhance the scalability and clinical applicability of the framework. Another promising direction involves integrating multimodal neuroimaging data (e.g., structural and diffusion MRI) to provide a more comprehensive representation of brain structure and function, which could further improve diagnostic performance. Additionally, evaluating the model on larger and more diverse cohorts will be crucial for validating its generalization ability and robustness across different populations and acquisition sites. Future work could investigate the contribution and stability of individual features through robustness tests or perturbation-based analyses, providing deeper insight into the interpretability and generalizability of the proposed method.

## 5. Conclusions

In summary, this work presents a novel multi-atlas guided multi-view contrast learning for ASD classification that effectively captures both local and global subject features while mitigating the effects of data heterogeneity. By leveraging contrastive alignment and consistency constraints, the proposed framework achieves improved performance and interpretability compared to existing methods. Although several challenges remain, the insights and methodologies developed here lay a solid foundation for future research on robust and scalable brain network analysis in neurodevelopmental disorder diagnosis.

## Figures and Tables

**Table 2:** Summary of demographics for ASD and TC groups across different sites.

Site	ASD		TC	
	Age	Male/Female	Age	Male/Female
NYU	14.9 ± 7.0	64/9	15.7 ± 6.2	72/26
UM	13.8 ± 2.3	39/9	15.0 ± 3.7	48/16
UCLA	13.3 ± 2.6	34/2	13.2 ± 1.8	33/5
USM	24.6 ± 8.5	38/0	22.3 ± 7.7	23/0
LEUVEN	18.0 ± 5.0	25/2	18.2 ± 5.0	29/5
PITT	19.4 ± 7.3	18/4	19.1 ± 6.2	20/3
TRINITY	17.0 ± 3.0	21/0	17.5 ± 3.6	23/0
YALE	13.0 ± 2.8	12/7	12.9 ± 2.8	18/7
MAX_MUN	30.4 ± 13.6	15/3	25.9 ± 8.1	23/1
KKI	9.6 ± 1.3	9/3	10.1 ± 1.1	19/7
CALTECH	27.4 ± 10.0	15/4	28.5 ± 10.7	13/4
STANFORD	10.2 ± 1.6	13/4	9.9 ± 1.6	15/4
SDSU	15.1 ± 1.6	12/0	14.3 ± 1.8	15/6
OLIN	16.8 ± 3.6	11/3	17.5 ± 3.0	9/2
SBL	35.3 ± 10.4	14/0	35.2 ± 5.4	11/0
OHSU	11.4 ± 2.1	12/0	10.4 ± 1.0	11/0
CMU	30.3 ± 6.9	3/0	25.5 ± 4.5	1/1
**Total**	17.7 ± 8.9	355/50	16.8 ± 7.4	383/87

**Table 3:** Device parameters and model parameters.

Name	Detail
Development system	Ubuntu 18.04
RAM	755 GB
CPU	14 vCPU Intel(R) Gold 6348
GPU	A800-80GB
learning rate	0.0001
Epochs	200
Weight decay	5 × 10^−5^
Dropout	0.2
Edge dropout	0.3
Early stopping epoch	50
Early stopping patience	30
Chebyshev convolution layers	2
hidden layers	32

**Table 4:** Compare the classification performance of the methods.

Method	*ACC* (%)	*PRE* (%)	*RECALL* (%)	*F1* (%)	*AUC* (%)
Random Forest	57.98±1.02	59.49±1.11	76.80±2.76	67.02±1.05	55.60±1.11
SVM	62.32±2.84	65.60±2.84	68.15±2.07	66.83±2.12	61.59±3.04
ASD-DiagNet	60.55±2.51	64.39±2.03	57.74±2.45	42.26±3.27	64.28±1.15
ASD-SAENet	64.67±7.44	60.49±10.09	63.60±14.33	62.00±8.63	72.01±7.89
BrainGNN	60.41±2.61	55.61±2.19	71.38±1.82	62.59±1.09	63.19±1.42
Deep-GCN	72.40±2.83	73.05±3.44	80.63±3.12	75.97±1.79	79.76±3.40
BNT	66.80±3.54	66.97±3.46	66.47±3.50	66.44±3.35	71.69±3.77
FBNetGen	64.92±6.36	66.46±6.66	76.68±8.74	71.90±8.27	63.89±4.13
MVS-GCN	67.13±4.21	65.96±2.47	78.15±2.80	71.70±2.47	63.23±3.17
GCN-MDD	68.16±1.75	72.35±1.38	79.85±1.99	73.06±1.59	75.48±1.82
TP-MIDA	70.49±1.09	64.72±1.45	68.44±0.94	66.53±0.94	75.62±0.85
deepManReg	69.22±6.25	67.65±7.21	64.25±11.06	65.90±8.15	77.97±5.42
DG-DMSGCN	72.00±6.85	74.56±10.29	71.22±9.75	70.11±8.55	72.84±8.24
RGTNet	71.98±2.11	72.99±1.70	72.05±1.56	70.59±1.92	70.86±1.50
GBT	68.16±2.02	68.83±2.05	68.33±1.40	67.19±1.82	76.25±1.34
CcSi-MHAHGEL	78.54±2.71	82.08±4.02	74.27±1.55	77.03±2.34	87.12±2.92
**MAMVCL**	85.71±0.43	86.24±1.27	87.67±2.33	86.84±0.59	92.93±0.13

**Table 5:** The result of the ablation experiment.

Variants	TAA	GCN	GCL	*ACC* (%)	*PRE* (%)	*RECALL* (%)	*F1* (%)	*AUC* (%)
Variant-I	✓			68.21 ± 1.56	69.43 ± 1.29	76.33 ± 0.63	71.35 ± 1.15	75.23 ± 2.13
Variant-II		✓		77.46 ± 1.50	79.39 ± 1.19	81.41 ± 1.63	80.00 ± 1.25	87.44 ± 1.40
Variant-III	✓	✓		83.48 ± 0.86	86.55 ± 1.24	82.12 ± 1.13	84.08 ± 0.95	91.02 ± 1.34
**MAMVCL**	✓	✓	✓	85.71 ± 0.43	86.24 ± 1.27	87.67 ± 2.33	86.84 ± 0.59	92.93 ± 0.13

TAA: Target-aware attention aggregator module; GCN: Graph convolutional networks module; GCL: Graph contrast learning module.

**Table 6:** Effects of phenotypic information on model performance (%).

Phenotypic Information	*ACC* (%)	*PRE* (%)	*RECALL* (%)	*F1* (%)	*AUC* (%)
S	84.28 ± 0.30	84.71 ± 1.19	86.77 ± 1.12	85.58 ± 0.10	91.76 ± 0.43
A	69.23 ± 0.78	69.59 ± 1.13	78.36 ± 1.24	73.33 ± 1.05	74.27 ± 1.58
G	68.67 ± 1.62	71.91 ± 2.04	69.12 ± 2.44	70.22 ± 2.84	74.58 ± 2.33
S + A	85.71 ± 0.43	86.24 ± 1.27	87.67 ± 2.33	86.84 ± 0.59	92.93 ± 0.13
S + G	77.82 ± 1.35	78.00 ± 1.24	82.13 ± 1.54	79.91 ± 1.39	84.17 ± 1.46
A + G	68.82 ± 2.29	70.80 ± 0.53	74.78 ± 4.18	72.42 ± 2.12	75.64 ± 2.02
S + A + G	82.50 ± 1.39	83.08 ± 1.24	84.18 ± 0.74	83.55 ± 0.98	89.92 ± 1.12
S + A + G + E	80.68 ± 0.45	83.25 ± 1.03	78.34 ± 1.43	81.12 ± 1.09	87.43 ± 0.79
S + A + G + H	80.54 ± 0.52	82.97 ± 1.11	78.89 ± 1.36	81.35 ± 1.15	87.61 ± 0.83
S + A + G + E + H	79.02 ± 0.68	81.41 ± 1.59	78.12 ± 1.21	79.21 ± 1.02	86.28 ± 0.76
S + A + G + FVP	80.91 ± 1.24	83.12 ± 1.33	77.95 ± 1.41	80.86 ± 1.12	87.19 ± 0.81
S + A + G + E + H + FVP	80.85 ± 0.46	83.67 ± 1.05	78.41 ± 1.39	81.25 ± 1.07	87.55 ± 0.78

S: site; A: age; G: gender; E: eye state during resting-state scan; H: handedness; FVP: full-scale/verbal/performance IQ.

## Data Availability

The original data presented in the study are openly available in ABIDE I dataset at https://fcon_1000.projects.nitrc.org/indi/abide/abide_I.html (accessed on 3 August 2025).

## Abbreviations

The following abbreviations are used in this manuscript:

$X^{msa}$	Brain network representation after multi-head self-attention (MSA)
$X^{te}$	Brain network representation after the NCAA module
$X^{fc}$	Functional connectivity matrix computed from rs-fMRI data using Pearson correlation
$X^{ut}$	Node initial features extracted from the upper triangular elements of $X^{fc}$
*N*	Total number of subjects across all sites
*F*	Binarized adjacency matrix indicating whether an edge exists between two subjects
$F_{ij}$	Element of *F* representing the presence (1) or absence (0) of an edge between subjects
	*i* and *j*
*A*	Edge score matrix of the fully connected population graph
$A_{ij}$	Edge score between subject *i* and subject *j*
$\theta_{1}$	Hyperparameter controlling the sparsity of the population graph
$\theta_{2}$	Hyperparameter threshold controlling the similarity criterion for continuous
	phenotypic features
$N_{m}$	Number of phenotypic features used in similarity computation
*P*	Matrix of phenotypic features of all subjects
$P_{im}$	*m*-th phenotypic feature of subject *i*
φ(·)	Similarity function between two phenotypic features
*W*	Weighted adjacency matrix of the population graph after cosine similarity computation
$W_{ij}$	Weighted edge strength between subject *i* and subject *j*
Cos(·)	Cosine similarity function used to compute similarity between feature vectors
$G_{i}$	Membership vector of the *i*-th view after graph convolution learning
$T_{i}$	Membership vector of the *i*-th view after Target-aware attention aggregator layer
V	Set of all distinct views in the model
$L_{\mathrm{vc}}$	View-consistency loss function
$z^{lv}$	Representation vector output from the local-view branch
$z^{gv}$	Representation vector output from the global-view branch
$\phi^{lv}$	Projected representation of $z^{lv}$ in the shared latent space
$\phi^{gv}$	Projected representation of $z^{gv}$ in the shared latent space
Proj(·)	Non-linear projection head mapping representations into a shared space
$\mathrm{sim}(\phi_{i},\phi_{j})$	Similarity score between two projected representations $\phi_{i}$ and $\phi_{j}$
τ	Temperature parameter controlling the smoothness of similarity distribution
$M^{p}$	Positive sample mask used to indicate matching pairs in contrastive learning
$L_{lv}$	Contrastive loss from local-view to global-view representation
$L_{gv}$	Contrastive loss from global-view to local-view representation
$L_{cl}$	Final total contrastive loss combining $L_{lv}$ and $L_{gv}$
γ	Balancing coefficient between the two contrastive losses
σ(·)	Sigmoid activation function
